# A speculation on the tandem fasciclin 1 repeat of FLA4 proteins in angiosperms

**DOI:** 10.1080/15592324.2018.1507403

**Published:** 2018-08-27

**Authors:** Aysegül Turupcu, Wisam Almohamed, Chris Oostenbrink, Georg J. Seifert

**Affiliations:** a Department of Material Sciences and Process Engineering, University of Natural Resources and Life Sciences, Vienna, Austria; b Department for Applied Genetics and Cell Biology, University of Natural Resources and Life Sciences, Vienna, Austria

**Keywords:** Fasciclin like arabinogalactan proteins, receptor like kinase, homology modelling

## Abstract

The *Arabidopsis thaliana Fasciclin like arabinogalactan protein 4 (FLA4)* locus is required for normal root growth in a linear genetic pathway with the *FEI1* and *FEI2* loci coding for receptor-like kinases. The two Fas1 domains of FLA4 are conserved among angiosperms but only the C-terminal Fas1 domain is required for genetic function. We show that at low salt deletion of the N-terminal Fas1 domain of transgenic *FLA4* leads to enhanced root elongation compared to the tandem Fas1 wild type version. Modeling the hypothetical interaction between FLA4 and FEI1 we show that the predicted interaction is predominantly involving the C-terminal Fas1 domain. Relative conformational mobility between the two FLA4 Fas1 domains might regulate the interaction with the FEI receptor kinases. We therefore speculate that the FLA4 FEI complex might be a sensor for environmental conditions in the apoplast.

## Results and discussion

**Figure 1:**
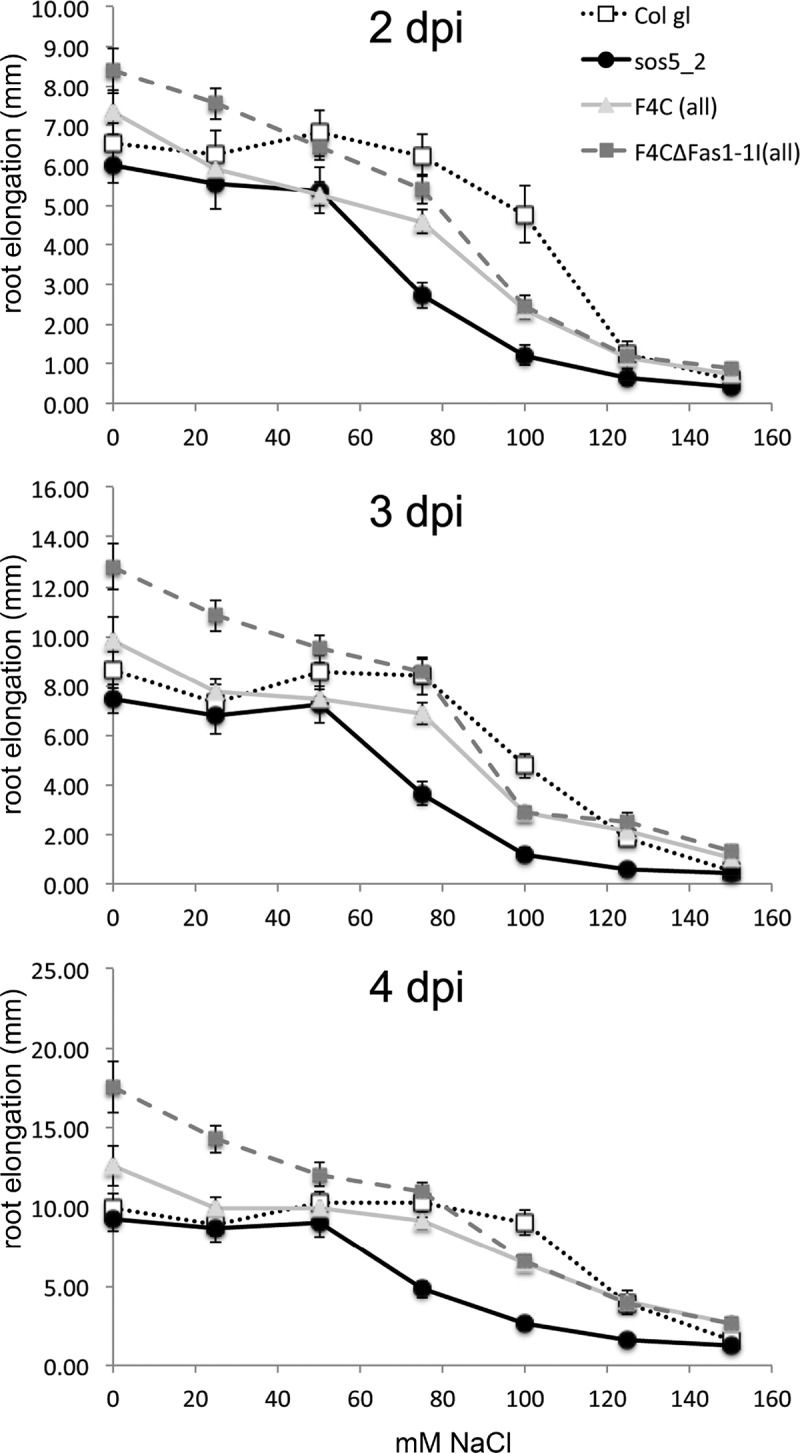
Root growth in Col gl (wild type), *sos5-1* (mutant) and *sos5-1* transformed with UBQ:*F4C* or UBQ:*F4C∆Fas1-1* constructs. Seedlings were transferred to semi-solid half-strength MS medium containing 0.5% sucrose and varying concentrations of NaCl. Root growth was determined 2 to 4 days after transfer (dpi). 20 seedlings per genotype and three independent transformed lines for each construct were tested. Confidence margins for α = 5% are shown.

The fasciclin 1 (Fas1) domain is a structural feature of many extracellular proteins in all clades of life and has been implicated with adhesion to and signalling influenced by the extracellular matrix.^1–5^ Many eukaryotic Fas1 proteins, including several groups of fasciclin like arabinogalactan proteins (FLAs) in plants, contain two or more Fas1 domains arranged in tandem. However, the biological role of Fas1 tandems is elusive. The FLA4 protein encoded by the *Arabidopsis thaliana Salt Overly Sensitive 5* (*SOS5)* locus is required for normal root growth and salt tolerance and acts in a linear genetic pathway with two leucine rich repeat receptor kinases encoding loci named *FEI1* and *FEI2*.^6–8^ Sequence searches suggest that every angiosperm genome contains at least one putative orthologue of *FLA4* containing both, the N-proximal Fas1-1 and the C-proximal Fas1–2 domain in tandem. Therefore, it was surprising that, in a study previously published in The Plant Journal,^9^ *FLA4* exerted its role for root growth depending exclusively on Fas1-2 and did not require Fas1-1 for its function. Both full-length *FLA4-citrin (F4C)* constructs as well as a *F4C* construct lacking the Fas1-1 (*F4C∆Fas1-1*) restored normal root growth to *sos5* mutants. In fact, in most experiments, when root growth on NaCl-free full-strength Murashige and Skoog medium (MS) was measured, independent *UBQ:F4C∆Fas1-1 (sos5-1)* lines not only complemented the growth defect of the *sos5-1* mutant background, but slightly exceeded the values of *UBQ:F4C(sos5-1)* lines or the Col gl wildtype. The difference between *UBQ:F4C(sos5-1)* and *UBQ:F4C∆Fas1-1 (sos5-1)* lines, however, was significant (P < 0.01) after prolonged growth periods on half strength MS medium containing no or only low concentrations of NaCl (Figure 1). This suggests that at low salt concentrations, Fas1-1 plays a negative role for the dominant growth stimulatory role of FLA4 Fas1-2 in root growth. By contrast, when the NaCl concentration exceeded 50 mM, the full length *UBQ:F4C(sos5-1)* and the truncated *UBQ:F4C∆Fas1-1 (sos5-1)* lines behaved identically which indicates that under these conditions, the N-terminal Fas1-1 domain is neutral for the role of FLA4 in root growth.

Is there a structural-mechanistic explanation for this observation? Because no crystal structures of FLA4 or FEI RLKs are available, we approached this question using molecular modelling. One of the crystal structures of Fas1 domains in the Protein Data Bank belongs to human transforming growth factor-beta-induced protein (TGFBIp) having four Fas1 domains (PDB ID 5NV6,^10^) and another one shows the Fas1-3 and Fas1-4 domains of *Drosophila* fasciclin I (PDB ID 1O70,^11^). We used SwissModel to make a homology model of FLA4 using these two crystal structures as a template.^12–14^ The FLA4 sequence has a higher identity with TGFBIp than Drosophila fasciclin I with 21.6% and 17.4%, respectively (the input sequence for the Swissprot query and a list of available structures including sequence identity and GMQE and is presented in the online supplement). Furthermore, although both models do not have a high global model quality estimation score (GMQE: 0.5), the model built with TGFBIp has a better agreement between the model structure and experimental structures. This ‘degree of nativeness’ is represented by the QMEAN score which is higher in the model based on TGFBIp with a score of −4.6 compared to model with Drosophila fasciclin I of −5.3. For these reasons, we continued with the model that was based on the TGFBIp structure. Next, a 3D molecular model of FLA4 with glycosylation at eight N-glycosylation sites was made. Representative glycan structures were added from a library created through enhanced sampling to get a large variety of conformers, as explained in reference.^15^ As a putative interaction partner, a homology model of the extracellular domain of FEI1 was modelled based on the structure of the extracellular domain of Brassinosteroid Insensitive 1- Associated Kinase 1 (BAK1, PDB code 4MN8;^16^ 32.6% sequence identity with FEI1, corresponds to LRR 1–5 domains).

**Figure 2:**
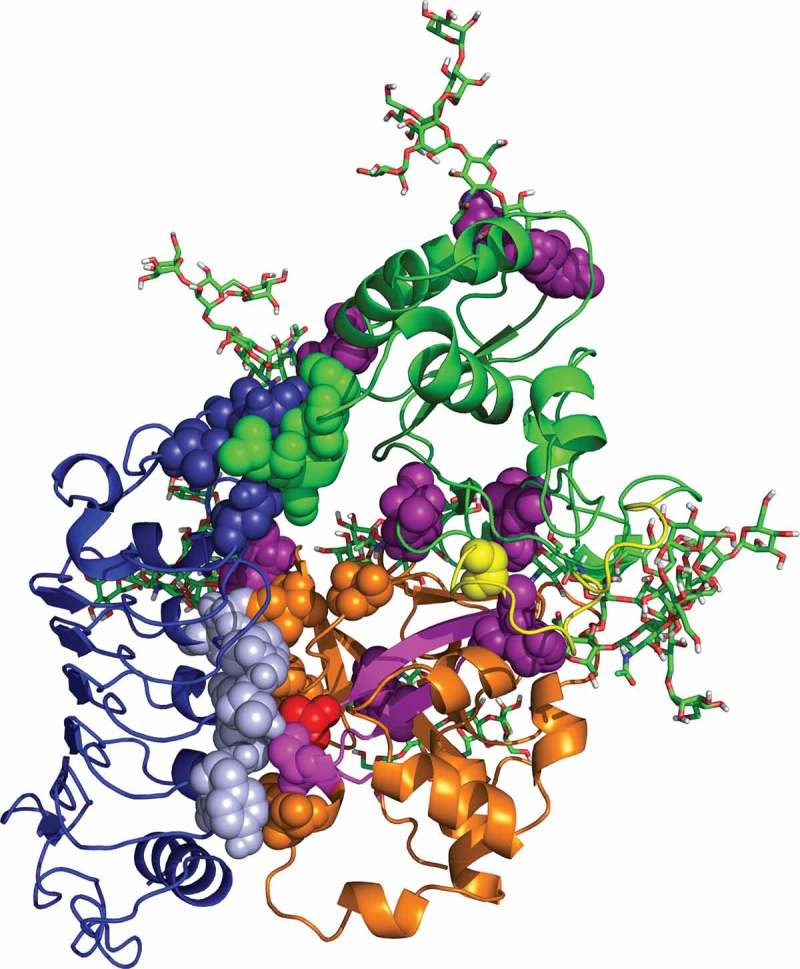
FLA4 – FEI1 interaction model. Homology model of FLA4 protein having flexible linker (coloured in yellow) connecting the Fas1-1 (green) and Fas1-2 (orange) domains. N- glycosylation was modelled at positions 30, 40, 135, 154, 167, 207, 312 and 317 using short high-mannose glycan structures for representation. Corresponding ASN residues colored in purple are represented as spheres. The predicted interaction face between FEI1 (coloured in blue) and Fas1-2 is shown as light blue and orange spheres, respectively. Interface residues between FEI1 and Fas1-1 domain are coloured in dark blue and green. Conserved residue SER348 coloured in red is substituted in the full loss of function allele *sos5-1*.^6^ Fas1-2 domain hotspots SER251, SER254, ASP255, VAL316, ASN317, ILE320, LEU329, ALA330, THR333, SER348 are shown as orange spheres. Their interaction partners on FEI1 PRO89, TYR111, GLY112, ALA113, THR116, THR135, GLY136, PRO137, ALA140, GLU141 are light blue spheres. Fas1-1 domain hotspots are PHE73, SER74, ALA76, SER77, LEU78 and THR81 shown as green spheres. Their interaction partners on FEI1 are ASN205, VAL213, CYS215 and GLN216 (dark blue spheres, this part of FEI1 corresponds to the beginning of the model, and shows no secondary structure in the model).

We next generated a model for the hypothetical protein-protein interaction between FEI1 and FLA4^7^ using a knowledge based protein interaction prediction tool, PRISM.^17^ PRISM predictions use the observation that different protein structures can interact through shared motifs. First, PRISM predicts binding residues by using structural and evolutionary similarity to known protein interfaces as a template set, and then it performs a flexible docking with a fiberdock energy function, producing negative values for strong interactions and positive values for poor interactions.^18^ After the predicted structural similarity is compared with the geometrical alignment, a possible interaction is proposed. Conserved hot spots at the interface are identified as the residues having evolutionary similarity. Using our homology models for FLA4 and FEI1 as an input to predict possible interaction two predictions were suggested with two different interfaces; 1x77AB and 3shoBC. The first interaction model yielded −9.41 fiberdock score representing the larger interface between FEI1 and Fas 1-2 (Figure 2). The second interface related to the smaller interaction between FEI1 and Fas1-1 which corresponded to the beginning of the homology model of FEI1 that has no secondary structure and has only few hotspots on the interface. Hence we believe the Fas1-2 interaction to be the more likely one (Figure 2). The interface contacts for each residue are summarized in Table 1.

**Table 1.** Interface residue contacts between FLA4 and FEI1. Two hydrogen bond pairs in the Fas1-2 domain are marked with asterisk.

Fas1-1 Domain	FEI
PHE 73	GLN 216
SER74	GLN 216, CYS 215
ALA 76	GLN 216
SER 77	ASN 205, GLN 216
LEU 78	ASN 205, GLN 216, VAL 213
THR 81	GLN 216
**Fas1-2 Domain**	**FEI**
SER 251*	THR 135*, GLY 112
SER 254	TYR 111
ASP 255	PRO 89, GLY 112
VAL 316	GLU 141
ASN 317	ALA 140
ILE 320	ALA 140
LEU 329	THR 116
ALA 330*	THR 116*
THR 333	GLY 136, PRO137
SER 348	ALA 113

**Figure 3.**
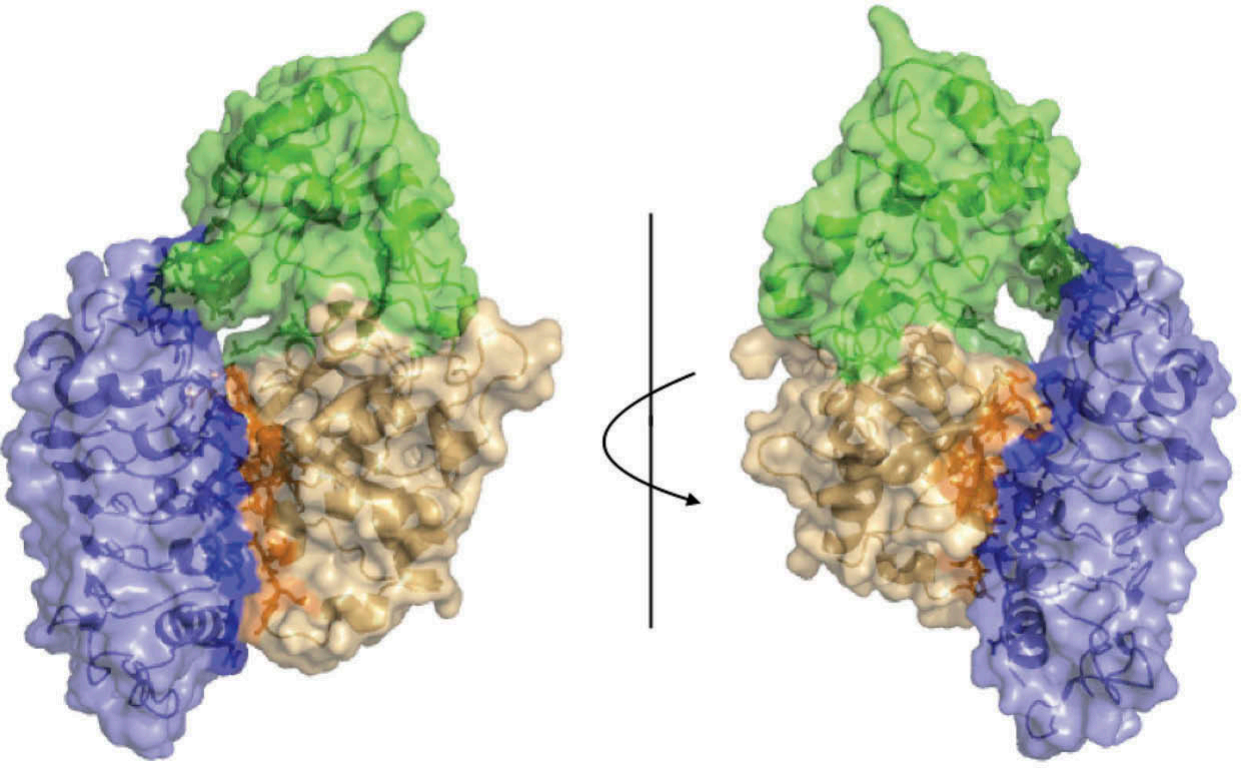
Surface representation of the proposed interaction model of FLA4-FEI (blue) from front view (left) and back view (right). Fas1-1 and Fas1-2 shown in green and orange respectively. Interface residues are shown darker with sticks.

The relatively weak support for an interaction with the Fas1-1 domain concurs with the recent observation that Fas1-1 was not essential for complementation.^9^ By contrast, the interaction model between FEI1 and Fas1-2 showed multiple interaction sites. To understand the driving force of the specific interaction, it is important to determine the characteristics of the protein interfaces. Physical and chemical features of the proposed interface were investigated in terms of hydrophobicity, electrostatic interactions, interface size and the occurrence of hydrogen bonds. In Table 1, residue contacts at the interface of the proposed complex between FLA4 and FEI1 is given for both FLA4 domains (Fas1-1 and Fas1-2) individually. As can be seen from both Figure 3 and Table 2, the interface between Fas1-2 and FEI1 is larger than the interface with the Fas1-1 domain. Furthermore, there are two hydrogen bond pairs at the Fas1-2 and FEI1 interface, reported in Table 1. The PDBePISA webserver^19^ was used to obtain the solvation free energy gain upon interface formation (Δ^i^G, in kcal/mol) reported in Table 2 along with the accessible and buried surface area of the interface. It can be seen from Table 2 that the Fas1-2 Domain and FEI1 interface has higher accessible surface area, which is an important structural characteristic during the formation of the protein-protein interaction. Also, it has Δ^i^G = −9.8 kcal/mol, indicating a hydrophobic interface.

**Table 2.** Characteristics of the interface between proposed interaction of the FLA4 – FEI model.

	ASA* (${A^{\circ }}^{2}$)	BSA* (${A^{\circ }}^{2}$)	Δ^i^G (kcal/mol)
Fas1-1 Domain & FEI1	7322 & 1700	284 & 281	−2.3
Fas1-2 Domain & FEI1	8086 & 7173	568 & 651	−9.8

*ASA: Accessible Surface Area, **BSA**: Buried Surface Area, **Δ^i^G**: Solvation energy effect

While some LRR ligands such as flagellin bind to the ascending lateral (convex) surface of the LRR-domain,^20^ others such as the *Spätzle* morphogen^21^ or the follicle-stimulating hormone^22^ interact with the concave LRR surfaces of their respective receptors. In our model the convex surface of the FEI1 ectodomain binds to the surface of the Fas1-2 domain (Figure 3) that is formed by the outward bent β-sheet harbouring the Ser348 residue (marked red in Figure 2). Intriguingly, this residue is mutated to Phe in the strong *sos5-1* allele,^6^ adding genetic support to our structural model.

We suggest that the Fas1-1 domain might negatively regulate FLA4 Fas1-2 binding to FEI1. This arrangement allows for regulatory flexibility. The relative conformation between Fas1-1 and Fas1-2 might be affected by post-translational modification (e.g. N-glycosylation seen in Figure 2) and their post-secretory remodelling, by interactions with other macromolecules or by apoplastic factors such as salinity or pH. In combination with FEI RLKs FLA4 might be an environmental sensor. Based independently on genetic evidence and structural predictions we speculate that the conformational changes of the tandem Fas1 protein FLA4 might regulate its interaction with FEI1 (and FEI2) LRR-RLKs to control root growth. Under all conditions Fas1-2 is predicted to interact with FEI1 which might in turn control growth by interacting with the biosynthesis and perception of growth regulators.^7,23,24^ Under some conditions such as low salt concentration, this interaction might be negatively modulated in an auto-inhibitory fashion by the Fas1-1 domain.

**Figure 4.**
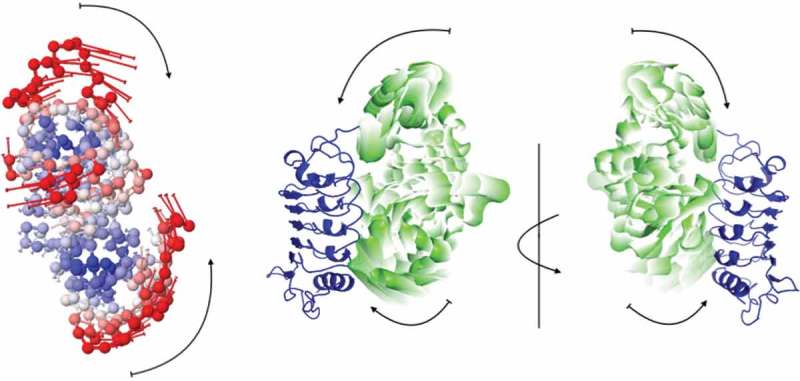
Predicted collective motion of FLA4 from Elastic Network Model. In the left panel, the FLA4 structure is colored according to the size of motions; red being most mobile and blue the most rigid. In the right panels, the motion is represented in the complex, where the transparency of the Fas1-1 and Fas1-2 domains (green) decreases, the closer to the FEI domain (blue) they are.

To gain an insight about the possible reconfiguration of FLA4, elastic network models (ENMs) were used. These models take the structure of a protein as a network taking atoms as nodes connected by springs. To predict the collective motion of FLA4, we used the Elastic Network Model server.^25–27^ The relative motion of the Fas1-1 and Fas1-2 domains are shown in the left panel of in Figure 4. Although the predicted motion is not causing a complete closing of the domains, rearrangement might affect the interaction of FEI as illustrated in the right panels of Figure 4.

Alternatively, Fas1-1 might prevent FLA4 from ectopically interacting with other receptor kinases. An auto-regulatory role, previously suggested for human TGFBIp,^2^ might be the basis for the evolution of Fas1 tandems in eukaryotes and for the conservation of the tandem Fas1 architecture of *FLA4* orthologues in angiosperms. Besides being fully consistent with available evidence, our speculative model provides testable predictions on the hypothetical physical interaction between these intriguing regulatory proteins.
